# Prevalence of *mecA, mecC* and *Panton-Valentine-Leukocidin* Genes in Clinical Isolates of Coagulase Positive Staphylococci from Dermatological Canine Patients

**DOI:** 10.3390/microorganisms10112239

**Published:** 2022-11-12

**Authors:** Marcela O. Platenik, Linda Archer, Lopamudra Kher, Domenico Santoro

**Affiliations:** Department of Small Animal Clinical Sciences, College of Veterinary Medicine, University of Florida, 2015 SW 16th Avenue, Gainesville, FL 32610, USA

**Keywords:** methicillin resistance, *mecA*, *mecC*, *pvl*, coagulase-positive staphylococci, multiplex PCR

## Abstract

Coagulase positive Staphylococci (*CoPS*) are the leading cause of canine cutaneous and otic infections. Virulence factors associated with Staphylococci include the expression of *mec* and *panton-valentine leukocidin (pvl)* genes. Methicillin-resistance (MR) is commonly associated with *mecA* gene expression, although a recently identified variant, *mecC*, has been reported. This study aims to evaluate the prevalence of *mecA*, *mecC* and *pvl* genes in 232 clinical isolates of *CoPS* collected from dogs with pyoderma. A multiplex PCR, and Kirby-Bauer disk diffusion susceptibility test for cefoxitin was performed for all isolates. PBP2a agglutination test was performed on 127 isolates. Standard MRSA isolates were used as positive controls. The *mecA* gene was identified in 149/232 isolates (64.2%): 116 *S. pseudintermedius*, 30 *S. coagulans* and three *S. aureus*. The *pvl* gene was present in only 1 isolate of *S. pseudintermedius* (0.4%), whereas no isolates carried the *mecC* gene. 34 isolates were resistant to cefoxitin (14.6%) and they were all *mecA* positive. The results of this study show an MR prevalence of 64.2% confirming concerns about antibiotic resistance in veterinary medicine. In conclusion, this is the first study analyzing the prevalence of *mecC* and *pvl* in comparison to *mecA*, in a large cohort of *CoPS* clinical isolates from dogs with pyoderma. A multimodal surveillance on the prevalence of *mecC* and *pvl* in veterinary medicine is essential to appropriate antimicrobial management.

## 1. Introduction

Staphylococci are resident bacteria and are a major constituent of the normal cutaneous and mucosal microbiota of humans and animals [1]. *Staphylococcus pseudintermedius* (*SP*) is the most common staphylococcal species isolated from the skin and mucosa of dogs. It can be isolated from the nares, mouth, pharynx, forehead, groin and anus of healthy companion animals [2,3]. *Staphylococcus pseudintermedius* is also the most common species associated with skin infections in dogs, although *S. coagulans* [4] (*SC*—previously known as *S. schleiferi sups. coagulans*), *S. schleiferi* (*SS*—previously known as *S. schleiferi sups. schleiferi*), and *S. aureus* (*SA*) are also frequently isolated [3,5]. *Staphylococcus coagulans* and *SS* have been reported to be associated with a number of infections in companion animals and humans, and they are becoming more prevalent in canine skin and aural infections [6]. Although it is a leading cause of infections in humans, *SA* is the least frequently isolated staphylococcal species in canine skin infection [7]. 

Methicillin-resistance (MR) is common among different staphylococcal species [8]. Unfortunately, many MR isolates are associated with multidrug resistance (MDR), defined as resistance to at least three antimicrobial classes [9], making therapeutic options very limited in clinical practice. Methicillin-resistant *Staphylococci* are generally characterized by the presence of the *mecA* gene that encodes the production of a low-affinity penicillin binding protein (PBP) known as PBP2a or PBP2′ [10]. This altered protein confers resistance to all β-lactam antibiotics, and their derivatives, including penicillins, and cephalosporins [11,12]. The *mecA* gene is located on a chromosomal mobile element, the staphylococcal chromosomal cassette (*SCCmec*), that can be transferred between different staphylococcal species [13]. In 2007, a new *mec* gene was recognized on the same mobile element (*SCCmec XI*) This *mec* homologue was initially referred to as *mecA*_LGA251_. The *mecC* gene in *S. aureus* is a part of the class E *mec* gene complex (*blaZ-mecC-mecR1-mecI*) (www.sccmec.org, accessed on 11 November 2022) and is commonly located on type XI SCC*mec* elements holding the *mecA*, later on identified as *mecC* [14,15]. The gene sequence of *mecC* is about 69% identical to that of *mecA*, and the protein (PBP2a/2′ also known as PBP2c) coded from *mecC* is about 63% identical to PBP2a at the amino acid level. This low level of identity between *mecA* and *mecC*, associated with the in vitro susceptibility to oxacillin for this latter, allows *mecC* organisms to not be detected by routine diagnostic tests (i.e., oxacillin microbroth dilution test, PBP2a agglutination test, and molecular identification of *mecA*) [14]. The majority of *mecC* MRSA demonstrates resistance to cefoxitin, and are therefore reported as MRSA. In contrast strains with *mecC* methicillin resistance demonstrate susceptibility to oxacillin [15], and may not be reported as MRSA. In particular, the phenotypical susceptibility to oxacillin and the PBP2a specific agglutination test for *mecC*-positive *Staphylococci* are of significant clinical relevance, being these the most commonly implemented identification tests for MR *Staphylococci*. In fact, although the identification of *mecA* (via polymerase chain reaction—PCR) is the gold standard, in the clinical practice, MR status in staphylococcal isolates is routinely assessed via the sensitivity to oxacillin and/or the expression of PBP2a [15,16]. Although, not routinely implemented, PCR has been recognized as the best way to identify *mecC* positive *Staphylococci* [15,16]. However, this methodology is not widespread, and the susceptibility to cefoxitin has been used as a suitable alternative [17]. 

Along with MR status, Panton-Valentine Leukocidin carrying *S. aureus* are more virulent and highly transmissible strains [18]. PVL is a member of a toxin family known as synergohymenotropic toxins, since they act on cell membranes by the synergy of two proteins that form cell pores, particularly targeting white blood cells, monocytes and macrophages [18,19]. Due to the significant cytotoxicity that PVL inflicts on the host, human skin and soft tissue infections caused by PVL-positive *SA* are usually more severe and have a more fulminant compared to when this virulence factor is absent [18]. 

After the first report of three *mecC*-*MRSA* isolates cultured from dairy cows in 2010, *mecC*-positive *Staphylococci* have been reported in cats, dogs, dairy cows, dairy and pet goats, and humans [17]. Regardless, the prevalence of *mecC* and *pvl* in dogs remains largely unknown. Recently, a study by Wu et al. [20] showed the lack of presence of *mecC* in 56 banked canine isolates of *SP* (unknown tissue source) [20]. However, no studies have been published on the simultaneous detection of *mecA*, *mecC* and *pvl* in canine isolates of coagulase positive *Staphylococci* (*CoPS*). Thus, the main aim of this study was to evaluate the prevalence of *mecA*, *mecC* and *pvl* genes in clinical isolates of CoPS (*SP, SC,* and *SA)* collected from dogs with pyoderma. The secondary aim of this study was to improve the ability to correctly identify MRSP, MRSA and MRSC (*mecA* and/or *mecC* positive) isolates in the canine population. To achieve these goals a multiplex PCR (mPCR), a variant of regular PCR, in which two or more loci are simultaneously amplified in the same reaction, was chosen as the most reliable and expeditious test for the detection of *mecA*, *mecC* and *pvl* genes [21]. A Kirby-Bauer disk diffusion test was also used to assess the susceptibility to cefoxitin [17].

## 2. Materials and Methods

### 2.1. Bacteria Isolates

A total of 232 *CoPS* clinical isolates were included in this study. In particular, 182 (78.5%) isolates of *SP*, 47 (20.3%) isolates of *SC* and three (1.2%) isolates of *SA* obtained from dogs with pyoderma, seen at the authors’ institution between July 2019 and June 2020 were included. The identification of the genus and the species of each isolate was determined based on colony morphology, biochemical features, using the Trek Sensititre (TREK Diagnostic Systems, Inc.; Cleveland, OH, USA) automated system, according to Clinical and laboratory Standards Institute (CLSI) guidelines [22,23,24], and via matrix-assisted laser desorption/ionisation time-of-flight (MALDI-TOF) mass spectrometry with an Autoflex system (Bruker, Billerica, MA, USA) using the direct protocol according to the manufacturer’s instructions. The identity of *SC* was also assessed with a urease test (Urease test tablets^®^, Hardy Diagnostics, Santa Maria, CA, USA). Once characterized and identified, the isolates were stored in glycerol media at −80 °C until use. Some of the isolates (127/232) were tested for PBP2a via the latex agglutination test (MASTALEX™ MRSA, Hardy Diagnostics, Santa Maria, CA, USA). Bacterial DNA was extracted (see next section) from all the isolates and from two reference, commercially available (American Type Culture Collection, Manassas, VA, USA), isolates of MRSA (ATCC 1756, carrying both *mecA* and *pvl* genes, and ATCC 2312, carrying the *mecC* gene). 

### 2.2. Multiplex Polymerase Chain Reaction (mPCR)

Multiplex PCR is a variant of the regular PCR in which two or more loci are simultaneously amplified in the same reaction [25]. The frozen clinical isolates were subcultured on Columbia blood agar with 5% sheep blood (Remel, Lenexa, KS, USA) at 37 °C for 24 h. Then, a few colonies were collected and the genomic DNA was extracted using the NucleoSpin^®^ microbial DNA kit (Macherey Nagel, Duren, Germany) following the manufacturer’s instructions. DNA at a final concentration of 75 ng/µL was used as the template. The mPCR was performed in a final 25 µL volume reaction according to the manufacturer’s instructions (Qiagen Multiplex PCR kit; Qiagen, Hilden, Germany). The assay was performed in a thermal cycler (StepOnePlus^TM^ Real Time PCR System; Thermo Fisher, Waltham, MA, USA) according to the following the protocol: initial denaturation (95 °C, 5 min), 35 cycles each composed of denaturation (95 °C, 30 s), primer annealing (60 °C, 90 s) and extension (72 °C, 90 s) and a final extension (68 °C, 10 min). The primers used for this study are listed in Table 1.

**Figure 1:**
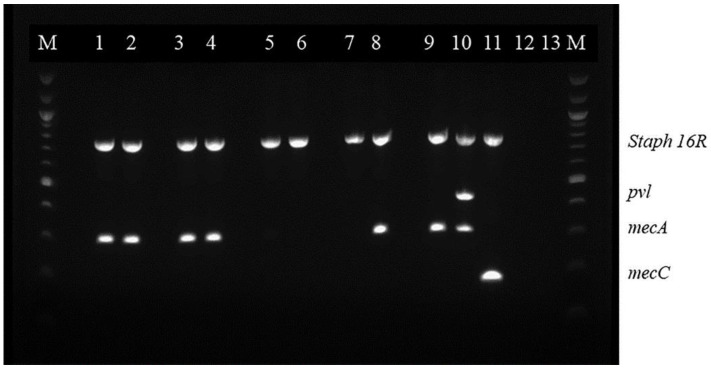
Multiplex PCR for coagulase positive staphylococci. M—Promega^®^ 100 BP DNA Ladder; Lanes 1–9 tested isolates; 10, MRSA (ATCC 1756) positive control for *Staph 16R*, *pvl* and *mecA*; 11, MRSA (ATCC 2312) positive control for *Staph 16R* and *mecC*; 12–13, negative controls.

**Figure 2:**
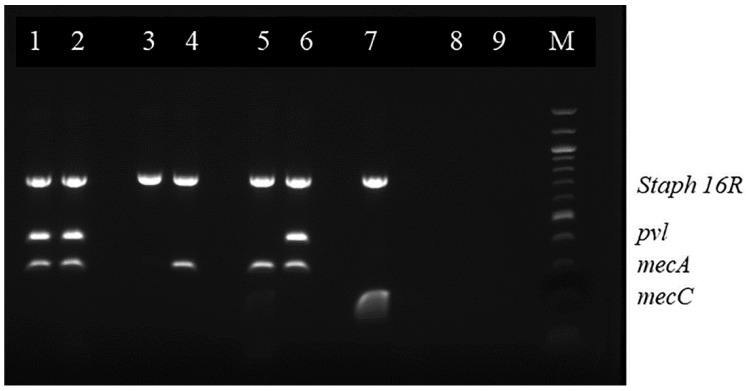
Multiplex PCR for coagulase positive staphylococci. M—Promega^®^ 100 BP DNA Ladder; Lanes 1–5 tested isolates; 6, MRSA (ATCC 1756) positive control for *Staph 16R*, *pvl* and *mecA*; 7, MRSA (ATCC 2312) positive control for *Staph 16R* and *mecC*; 8–9 negative controls.

The PCR-amplified samples were analyzed by agarose gel electrophoresis by using a horizontal 1% agarose gel in lx Tris/Borate/EDTA (TBE) buffer (pH 8.3; 0.09 M Tris, 0.09 M boric acid, 2.0 mM EDTA) and with 0.003% (wt/vol) ethidium bromide incorporated for DNA staining. 25 µL of sample was applied to each well and the gels were run at 150 V for 2 h. The PCR products were then visualized and photographed on a trans illuminator Fluor chem E (Sigma, St. Louis, MO, USA). Positive controls for *mecA* (ATCC 1756), *mecC* (ATCC 2312) and *pvl* (ATCC 1756) genes and negative control (master mix solution, primers and DNase/RNase-free distilled water) were also used in each gel. A *Staphylococcus*-specific region of the 16S rRNA gene was used as a positive internal control (Qiagen, Germany). A molecular weight marker 100-bp ladder (Promega^®^ 100 bp DNA Ladder Molecular Weight marker (Promega, Madison, WI, USA) was included on each gel. To validate the mPCR results, the DNA present in the bands corresponding to *mecA* and *pvl* genes, in the clinical isolates and the positive controls, as well as the *mecC* band in the positive control were extracted (QI Aquick gel extraction kit) and sequenced (GENEWIZ^®^, South Plainfield, NJ, USA) (Qiagen, Germany) [26,27] (Figure 1 and Figure 2).

### 2.3. Disk Diffusion Susceptibility Tests (Kirby-Bauer Test)

According to the CLSI guidelines [22], the Mueller-Hinton agar (Sigma-Aldrich^®^, St Louis, MO, USA) was aseptically inoculated with the clinical isolates, and the MRSA controls, at a concentration of 0.5 McFarland tested using a nephelometer (DEN-1B, Grant USA Inc., Beaver falls, USA). Then, a cefoxitin 30 µg disk was placed at the center of the plate gently positioned on the agar [23]. The plates were incubated at 37 °C for 24 h as per CLSI guidelines. A zone of inhibition was observed and measured to determine the susceptibility to the antibiotic based on CLSI guidelines for *CoNS* [24]. Measuring the diameter across the zone of inhibition over the center of the disk, an isolate would be classified as resistant (R), when a zone of inhibition was ≤24 mm, or susceptible (S) when a zone of inhibition was ≥25 mm.

### 2.4. Ethics Statement

This study does not involve animals. The microorganisms were banked via the clinical microbiology laboratory.

## 3. Results

In the group of 232 *CoPS* isolates tested in this study, the presence of the *mecA* gene was found in 149 (64.2%) of them. More specifically, *mecA* was present in 116/182 isolates (63.7%) of *SP*, 30/47 isolates (63.8%) of *SC* and 3/3 isolates (100%) of *SA.* The *pvl* gene was found only in 1 *SP* isolate representing the 0.4% and the 0.55% of *CoPS* and *SP* isolates, respectively. On the contrary, the *mecC* gene was not found in any isolate tested (Table 2).

Using the disk diffusion assay, 34/232 (14.7%) *CoPS* isolates were classified as resistant to cefoxitin. More specifically, 32/182 (13.8%) isolates of *SP*, 1/47 (2.1%) isolate of *SC*, and 1/3 (33.3%) isolate of *SA* were resistant to cefoxitin. All the isolates classified as resistant to cefoxitin were also *mecA* positive. The single *SP* isolate carrying the *pvl* gene was also *mecA*-positive and cefoxitin resistant. 

Furthermore, when the *mecA* positive isolates were cross-checked with the phenotypic characteristics of resistance, 31/149 (20.8%) isolates were resistant to oxacillin and cefoxitin, 81/149 (54.4%) were resistant to oxacillin and susceptible to cefoxitin, 1/149 (0.7%) isolate was susceptible to oxacillin and resistant to cefoxitin, and 37/149 (24.8%) isolates were susceptible to both oxacillin and cefoxitin. Details on the resistant patterns for each staphylococcal species are reported in Table 2.

## 4. Discussion

This is the first study analyzing the simultaneous detection of *mecA*, *mecC* and *pvl* in *CoPS* from canine patients with pyoderma. The most common bacterial species isolated in this study was *SP*, followed by *SC,* whereas *SA* was the least common staphylococcal species. The results of this study are not surprising since *SP* is the most common pathogen isolated in almost 90% of canine pyoderma [15]. The present study reveals the presence of the *mecA* gene in 64% of the *CoPS* included in this study, whereas *mecC* was not identified in any isolates and only 1 *SP* isolate carried the *pvl* gene. Furthermore, an overall resistance to cefoxitin was seen in 14.7% of the tested isolates. These data are in agreement with previous epidemiological studies showing an increased incidence of MR in *CoPS* isolated from canine patients with *SP* being the most commonly isolated pathogen [28]. The increased incidence of MRSP in dogs presents a major treatment challenge for veterinarians [20]. The results of this study also highlight the high incidence of MRSC in canine pyoderma with 30/47 isolates of *SC* being MR for an overall prevalence of 63.9% comparable to MRSP. As for MRSP, a potentially increased frequency of MRSC is of significant public health concern worldwide because of the involvement of such organisms in soft tissue and skin infections in companion animals and humans [1,12]. Dogs are not typically colonized by *SA*, however transient associations that can potentially lead to severe infections have been reported [6]. In the present study, only three *SA* isolates were collected, and all of them resulted to carry the *mecA* gene. Although these data support the high incidence of MRSA in canines, they need to be cautiously interpreted because of the very low number of isolates tested. 

When the presence of the *mecA* gene was compared to PBP2a coagulation test positivity (n = 127), in 117/127 (92.1%) isolates the two tests were in agreement with each other. The remaining ten isolates (7.9%) (4 *SP* and 6 *SC*) were positive to *mecA* and negative to the agglutination test confirming a high level of concordance between *mecA* expression and a positive PBP2a agglutination test. The discordance that was observed between the *mecA* gene presence and PBP2a agglutination test results may have several explanations. Recently, it has been hypothesized that some methicillin-susceptible organisms produce an inactive PBP2a translated from a truncated *mecA* gene [7]. In this case, a negative *mecA* PCR could be associated with a positive PBP2a agglutination test. On the contrary, MR isolates that are positive for *mecA* gene could contain a full-length copy of the gene, but they fail to express the PBP2a as a result of a mutation in the open reading frame or promoter, showing negative results on PBP2a test [7]. Finally, due to the heterogenous expression of the *mecA* gene, it is possible that because of the paucity of cells producing PBP2a, this can be missed on the regular latex agglutinaiton test [29]. To avoid false negative results, an overnight induction, in the presence of oxacillin, before testing may be necessary for an accurate identification of methicillin resistance, as it has been reported for coagulase negative staphylococci [29].

Furthermore, from the cohort of 149 *mecA* isolates of *SP*, *SC* and *SA*, 38 isolates (25.5%) were reported to be oxacillin susceptible based on the CLSI guidelines [21,30]. This means that although oxacillin is considered the most accurate test to identify *MRSP* some isolates would be misclassified as susceptible, while they are indeed resistant to β-lactam antibiotics. These results are in agreement with Wu et al. [20], pointing out how an accurate identification may be a challenge to laboratories that do not perform more than one methodology for identification of *mec* genes relying only on one test [20]. This is also the case for isolates carrying the *mecC* gene, routinely classified as susceptible based on oxacillin susceptibility and/or PBP2a agglutination test presenting a potential diagnostic challenge. Although no isolates tested positive for *mecC* in this cohort, larger studies are needed to confirm the results presented here. 

Cefoxitin is currently used as a surrogate marker of MR status in *SA* isolates since it is a strong inducer of the *mecA* operon that is currently utilized to detect methicillin resistance [24]. However, it is not a good indicator of an MR status for *SP or SC*, for which oxacillin sensitivity seems to be a much better and reliable marker of resistance [12,20]. These data were confirmed by the present study in which only 34/149 (22.8%) of the *CoPS* isolates tested were resistant to cefoxitin being *mecA* positive. The discordance between cefoxitin susceptibility and the expression of *mecA* is also clinically relevant and although the reasons for this discrepancy are not completely clear, this phenomenon could be explained by the heterogeneous expression of *mecA*, leading to resistance to oxacillin and sensitivity to cefoxitin [31].

Although, few studies have been published on the frequency of *pvl* gene in MRSA isolates [10], there are no studies assessing the presence of *pvl* in canine *CoPS*. In the present study, only 1 isolate, an *SP* isolate, was found to carry the *pvl* gene with a prevalence of 0.4% of the *SP* tested. Interestingly enough, this isolate also carried the *mecA* gene. However, because of the very low incidence of *pvl* in the bacterial cohort studied, it is not possible to make any inference on the association between *pvl* and *mecA*. Similarly to *pvl*, information on the prevalence of *mecC* in *CoPS* is scarce, although an increased frequency of *mecC* has been identified in *MRSA* cases [14]. Only one study reported the absence of *mecC* in canine and human isolates of *CoPS* [20]. The results of the present study, with an increased number of isolates tested, similarly point toward an absent expression of *mecC* in canine staphylococci, with no isolates testing positive for *mecC*. 

The results of this study highlight the need for diagnostic tests able to test for multiple genes at one time. Detection of the *mecA* gene by PCR or gene probe or deletion of its gene product by immunologic methods are considered to be the gold standards for confirmation of *mecA*-mediated resistance in *Staphylococci* [2,12]. mPCR, as an alternative, is a rapid assay to detect four genes at a time, showing high sensitivity and specificity [10]. Besides being easy to perform and less time-consuming, it is a cost-effective methodology. However, it is imporatnt to condier that all the primers in the reaction must have similar melting temperature, allowing all gene amplifications to process with the same temperature program. This study confirmed the usefulness of this methodology for the simultaneous detection of multiple genes for the identification of virulence factors in *CoPS* [10]. 

The major limitation of this study was related to the primers used for the *mecC* evaluation and their compatibility with different *Staphylococcus* species. In the present study, the primers that were used were previously validated for *SA* [32]. Although the primers were able to correctly identify *mecC* in the *MRSA* controls, it is still possible that this set of primers may not be the best for *SP* or *SC* depending on the degree of homology of *mecC* across the different species. In addition, since *SP* exhibits high genetic variability, the occurrence of recombination events rather than mutations may also interfere with the mPCR results [33].

## 5. Conclusions

In conclusion, this study confirms the high frequency of MR in canine *CoP* staphylococcal isolates, which should increase clinicians’ concern and awareness regarding the use of antibiotics in veterinary medicine. This study also highlights the need for multiple testing methodologies to confirm the presence of resistance genes, since they can be missed in routine microbiological tests (e.g., oxacillin/cefoxitin sensitivity and PBP2a agglutination test). Furthermore larger epidemiological studies are needed to confirm the results reported in this study and verify the incidence of *mecC* and *pvl* in canine clinical isolates. Such studies are fundamental to increase the surveillance of the prevalence and the implications of the *mec* homologs and *pvl* genes. Such information is relevant regarding the clinical treatment decision-making process in order to practice appropriate antimicrobial stewardship.

## Figures and Tables

**Table 1:** Nucleotide sequences and sizes of PCR products of amplified genes.

Primer	5′–3″	Target Gene	Amplicon Length (bp)	Reference
*mecA*-for	TGAAAAATGATTATGGCTCAGGTACT	*mecA*	173	[16]
*mecA*-rev	CTGGAACTTGTTGAGCAGWGGTTCT	*mecA*		
*mecC*-for	ATCTCGCCTTGGCCATATCCTGAA	*mecC*	171	[16]
*mecC*-rev	TGCCCGCATTGCATTAGCATTAGG	*mecC*		
*pvl*-for	ACA CAC TAT GGC AAT AGT TAT TT	*Pvl*	868	[26]
*pvl*-rev	AAA GCA ATG CAA TTG ATG TA	*Pvl*		
Staph756F	AACTCTGTTATTAGGGAAGAACA	16S	756	[22]
Staph 750R	CCACCTTCCTCCGGTTTGTCACC	16S		

**Table 2:** Phenotypic resistance to oxacillin and cefoxitin of mecA positive Staphylococcus strains.

MecA Positive Isolates	*Staphylococcus pseudintermedius*	*Staphylococcus coagulans*	*Staphylococcus aureus*
Oxacillin resistant/cefoxitin sensitive	54	26	0
Oxacillin sensitive/cefoxitin sensitive	31	3	2
Oxacillin resistant/cefoxitin resistant	30	1	1
Oxacillin sensitive/cefoxitin resistant	1	0	0
Total	116	30	3

## Data Availability

Not applicable.

## References

[1] Morris D.O., Rook K.A., Shofer F.S. (2006). Screening of *Staphylococcus aureus*, *Staphylococcus intermedius*, and *Staphylococcus schleiferi* isolates obtained from small companion animals for antimicrobial resistance: A retrospective review of isolates (2003–04). Vet. Dermatol..

[2] van Duijkeren E., Catry B., Greko C., Moreno M.A., Pomba M.C., Pyörälä S., Ruzauskas M., Sanders P., Threlfall E.J., Torren-Edo J. (2011). Review on methicillin-resistant *Staphylococcus pseudintermedius*. J. Antimicrob. Chemother..

[3] Han J.-I., Rhim H., Yang C.-H., Park H.-M. (2018). Molecular characteristics of new clonal complexes of *Staphylococcus pseudintermedius* from clinically normal dogs. Vet. Q..

[4] Madhaiyan M., Wirth J.S., Saravanan V.S. (2020). Phylogenomic analyses of the *Staphylococcaceae* family suggest the reclassification of five species within the genus *Staphylococcus* as heterotypic synonyms, the promotion of five subspecies to novel species, the taxonomic reassignment of five Staphylococcus species to *Mammaliicoccus* gen. nov., and the formal assignment of *Nosocomiicoccus* to the family *Staphylococcaceae*. Int. J. Syst. Evol. Microbiol..

[5] Rana E.A., Islam M.Z., Das T., Dutta A., Ahad A., Biswas P.K., Barua H. (2022). Prevalence of coagulase-positive methicillin-resistant *Staphylococcus aureus* and *Staphylococcus pseudintermedius* in dogs in Bangladesh. Vet. Med. Sci..

[6] Paterson G.K. (2021). Genomic epidemiology of the opportunistic pathogen *Staphylococcus coagulans* from companion dogs. J. Med. Microbiol..

[7] Bemis D.A., Jones R.D., Hiatt L.E., Ofori E.D., Rohrbach B.W., Frank L.A., Kania S.A. (2006). Comparison of tests to detect oxacillin resistance in *Staphylococcus intermedius*, *Staphylococcus schleiferi*, and *Staphylococcus aureus* isolates from canine hosts. J. Clin. Microbiol..

[8] Pietrocola G., Gianotti V., Richards A., Nobile G., Geoghegan J.A., Rindi S., Monk I.R., Bordt A.S., Foster T.J., Fitzgerald R. (2015). Fibronectin Binding Proteins SpsD and SpsL Both Support Invasion of Canine Epithelial Cells by *Staphylococcus pseudintermedius*. Infect. Immun..

[9] Magiorakos A.-P., Srinivasan A., Carey R.B., Carmeli Y., Falagas M.E., Giske C.G., Harbarth S., Hindler J.F., Kahlmeter G., Olsson-Liljequist B. (2012). Multidrug-resistant, extensively drug-resistant and pandrug-resistant bacteria: An international expert proposal for interim standard definitions for acquired resistance. Clin. Microbiol. Infect..

[10] Velasco V., Sherwood J.S., Rojas-García P.P., Logue C.M. (2014). Multiplex Real-Time PCR for Detection of Staphylococcus aureus, *mecA* and Panton-Valentine Leukocidin (PVL) Genes from Selective Enrichments from Animals and Retail Meat. PLoS ONE.

[11] Zhang K., McClure J.-A., Elsayed S., Louie T., Conly J.M. (2005). Novel Multiplex PCR Assay for Characterization and Concomitant Subtyping of Staphylococcal Cassette Chromosome *mec* Types I to V in Methicillin-Resistant *Staphylococcus aureus*. J. Clin. Microbiol..

[12] Petersen A., Stegger M., Heltberg O., Christensen J., Zeuthen A., Knudsen L., Urth T., Sorum M., Schouls L., Larsen J. (2013). Epidemiology of methicillin-resistant *Staphylococcus aureus* carrying the novel *mecC* gene in Denmark corroborates a zoonotic reservoir with transmission to humans. Clin. Microbiol. Infect..

[13] Bardiau M., Yamazaki K., Ote I., Misawa N., Mainil J.G. (2013). Characterization of methicillin- resistant *Staphylococcus pseudintermedius* isolated from dogs and cats. Microbiol. Immunol..

[14] Loncaric I., Kübber-Heiss A., Posautz A., Ruppitsch W., Lepuschitz S., Schauer B., Feßler A.T., Krametter-Frötscher R., Harrison E.M., Holmes M.A. (2019). Characterization of *mecC* gene-carrying coagulase-negative *Staphylococcus* spp. isolated from various animals. Vet. Microbiol..

[15] Paterson G.K., Harrison E.M., Holmes M.A. (2014). The emergence of *mecC* methicillin-resistant *Staphylococcus aureus*. Trends Microbiol..

[16] Paterson G.K., Morgan F.J.E., Harrison E., Cartwright E.J.P., Torok E., Zadoks R.N., Parkhill J., Peacock S.J., Holmes M. (2014). Prevalence and characterization of human *mecC* methicillin-resistant *Staphylococcus aureus* isolates in England. J. Antimicrob. Chemother..

[17] Brown D.F.J. (2001). Detection of methicillin/oxacillin resistance in staphylococci. J. Antimicrob. Chemother..

[18] Karmakar A., Jana D., Dutta K., Dua P., Ghosh C. (2018). Prevalence of Panton-Valentine Leukocidin Gene among Community Acquired *Staphylococcus aureus*: A Real-Time PCR Study. J. Pathog..

[19] Johnsson D., Mölling P., Strålin K., Söderquist B. (2004). Detection of Panton–Valentine leukocidin gene in *Staphylococcus aureus* by LightCycler PCR: Clinical and epidemiological aspects. Clin. Microbiol. Infect..

[20] Wu M.T., Burnham C.-A.D., Westblade L.F., Bard J.D., Lawhon S.D., Wallace M.A., Stanley T., Burd E., Hindler J., Humphries R.M. (2016). Evaluation of Oxacillin and Cefoxitin Disk and MIC Breakpoints for Prediction of Methicillin Resistance in Human and Veterinary Isolates of *Staphylococcus intermedius* Group. J. Clin. Microbiol..

[21] Becker K., Larsen A.R., Skov R.L., Paterson G.K., Holmes M.A., Sabat A.J., Friedrich A.W., Köck R., Peters G., Kriegeskorte A. (2013). Evaluation of a Modular Multiplex-PCR Methicillin-Resistant *Staphylococcus aureus* Detection Assay Adapted for *mecC* Detection. J. Clin. Microbiol..

[22] CLSI (2010). Surveillance for Methicillin-Resistant Staphylococcus aureus: Principles, Practices, and Challenges, A Report..

[23] CLSI (2015). Performance Standards for Antimicrobial Disk and Dilution Susceptibility Tests for Bacteria Isolates form Animals.

[24] CLSI (2015). Performance Standards for Antimicrobial Susceptibility Testing.

[25] Sasaki T., Kikuchi K., Tanaka Y., Takahashi N., Kamata S., Hiramatsu K. (2007). Methicillin-Resistant Staphylococcus pseudintermedius in a Veterinary Teaching Hospital. J. Clin. Microbiol..

[26] GENEWIZ, USA. https://www.genewiz.com/en/Public/Services/Next-Generation-Sequencing/Standalone-NGS-Solutions.

[27] QIAGEN Multiplex PCR Kit Handbook. https://www.qiagen.com/us/resources/download.aspx?id=a541a49c-cd06-40ca-b1d2-563d0324ad6c&lang=en.

[28] Kawakami T., Shibata S., Murayama N., Nagata M., Nishifuji K., Iwasaki T., Fukata T. (2010). Antimicrobial Susceptibility and Methicillin Resistance in Staphylococcus pseudintermedius and Staphylococcus schleiferi subsp. coagulans Isolated from Dogs with Pyoderma in Japan. J. Vet. Med. Sci..

[29] Hussain Z., Stoakes L., Garrow S., Longo S., Fitzgerald V., Lannigan R. (2000). Rapid detection of mecA-positive and mecA-negative Staphylococci by an anti-penicillin binding protein 2a slide latex agglutination test. J. Clin. Microbiol..

[30] Los F.C.O., Randis T., Aroian R.V., Ratner A. (2013). Role of Pore-Forming Toxins in Bacterial Infectious Diseases. Microbiol. Mol. Biol. Rev..

[31] Savini V., Di Giuseppe N., Fazii P., D’Amario C., D’Antonio D., Carretto E. (2013). *Staphylococcus pseudintermedius* heterogeneously expresses the *mecA* gene. Vet. Microbiol..

[32] Seidel C., Peters S., Eschbach E., Feßler A.T., Oberheitmann B., Schwarz S. (2017). Development of a nucleic acid lateral flow immunoassay (NALFIA) for reliable, simple and rapid detection of the methicillin resistance genes *mecA* and *mecC*. Vet. Microbiol..

[33] González-Domínguez M.S., Carvajal H.D., Calle-Echeverri D.A., Chinchilla-Cárdenas D. (2020). Molecular Detection and Characterization of the *mecA* and *nuc* Genes From Staphylococcus Species (*S. aureus*, *S. pseudintermedius*, and *S. schleiferi*) Isolated From Dogs Suffering Superficial Pyoderma and Their Antimicrobial Resistance Profiles. Front. Vet. Sci..

